# A 4-plex Droplet Digital PCR Method for Simultaneous Quantification and Differentiation of Pathogenic and Non-pathogenic *Vibrio parahaemolyticus* Based on Single Intact Cells

**DOI:** 10.3389/fmicb.2020.01727

**Published:** 2020-08-12

**Authors:** Shuwen Lei, Xiaokui Gu, Wei Xue, Zhangquan Rong, Zhe Wang, Song Chen, Qingping Zhong

**Affiliations:** ^1^Key Laboratory of Biomaterials of Guangdong Higher Education Institutes, Department of Biomedical Engineering, Jinan University, Guangzhou, China; ^2^Guangdong Provincial Key Laboratory of Food Quality and Safety, College of Food Science, South China Agricultural University, Guangzhou, China; ^3^Guangdong Laboratory of Lingnan Modern Agriculture, South China Agricultural University, Guangzhou, China; ^4^Guangdong Shunde Innovative Design Institute, Foshan, China

**Keywords:** *Vibrio parahaemolyticus*, 4-plex ddPCR, qPCR, single intact cell, precise detection

## Abstract

*Vibrio parahaemolyticus* is a significant seafood-borne pathogen, leading to serious acute gastrointestinal diseases worldwide. In this study, a reliable 4-plex droplet digital PCR (ddPCR) was successfully established and evaluated for the simultaneous detection of *V. parahaemolyticus* based on *tlh*, *tdh*, *ureR*, and *orf8* in food samples using single intact cells. The targets *tlh* and *ureR* were labeled with 6-Carboxyfluorescein (FAM), and the targets *tdh* and *orf8* were labeled with 5’-Hexachlorofluorescein (HEX). Due to reasonable proration of primers and probes corresponding into the two fluorescence channels of the ddPCR detecting platforms, the clearly separated 16 (2^4^) clusters based on fluorescence amplitude were obtained. For better results, the sample hot lysis time and the cycle number were optimized. The results showed that the minimum number of “rain” and maximum fluorescence amplification were presented for precise detection in the condition of 25 min of the sample hot lysis time and 55 cycles. The sensitivity of this 4-plex ddPCR assay was 39 CFU/mL, which was in accordance with that of the conventional plate counting and was 10-fold sensitive than that of qPCR. In conclusion, the 4-plex ddPCR assay presented in this paper was a rapid, specific, sensitive, and accurate tool for the detection of *V. parahaemolyticus* including pandemic group strains and could be applied in the differentiation of *V. parahaemolyticus* in a wide variety of samples.

## Introduction

*Vibrio parahaemolyticus*, a gram-negative and halophilic foodborne bacterium, which was first discovered in 1953 and commonly found in marine or estuarine all over the world, has been a leading causal agent of acute bacterial gastroenteritis with consumption of raw, mishandled or undercooked seafood (Fujino et al., 1953; Baross and Liston, 1968; Krantz et al., 1969). Up to now, *V. parahaemolyticus* has been acknowledged as the foremost foodborne pathogen that has caused outbreaks of foodborne illnesses worldwide. Take some examples: during May to September 2013, an outbreak associating with *V. parahaemolyticus* resulted in 104 illnesses along the U.S. Atlantic Coast (Newton et al., 2014); In India, 56.8% samples from diarrhoeal cases were identified for *V. parahaemolyticus* (Guin et al., 2019); In China, the number of the foodborne poisoning outbreak due to *V. parahaemolyticus* was increased from 31.1% between 1991 and 2001 to over 70% between 1998 and 2013. According to the data from the China National Center for Food Safety Risk Assessment (CFSA), there were 4.95 million people infected by *V. parahaemolyticus* per year (Pang et al., 2017). Therefore, it is crucial to develop rapid, accurate, reliable, and convenient identification methods for pathogenic *V. parahaemolyticus.* On the other hand, most of *V. parahaemolyticus* strains isolated from the environment are non-pathogenic, only a small part of them can cause food poisoning outbreaks (Velazquez-Roman et al., 2014). Hence, classification and identification of the pathogenic and non-pathogenic strains are also necessary.

Most of the conventional quantitative and identifying techniques, such as colony counting, have presented certain drawbacks with their inherent slowness, lots of workload and materials (Velusamy et al., 2010). In contrast to the conventional methods, rapid detection method such as PCR is convenient, sensitive and enables overcoming the disadvantages. PCR can be categorized into conventional PCR, quantitative PCR (qPCR), and (ddPCR). The ddPCR is an emerging technology for the detection and quantification of single or multiple targets simultaneously (Ottesen et al., 2006; Tadmor et al., 2011). Compared with other PCR techniques, ddPCR is an endpoint and absolute quantification approach based on the limiting dilution and Poisson distribution, unlike analog qPCR that relies on rate-based measurements (Ct values) and calibration curves (Hindson et al., 2011). It is not only more sensitive but also more accurate than qPCR in the quantification of rare target molecules at low target copy numbers (Bahar et al., 2018). Besides, ddPCR is remarkably efficient and high-throughput for processing 96 samples at a time (Hindson et al., 2013).

Effective detection of pathogenic *V. parahaemolyticus* requires the identification of several specific targets. The target of the thermolabile hemolysin gene (*tlh*) is used for the identification of total *V. parahaemolyticus* (Taniguchi et al., 1986; Bej et al., 1999). In contrast, thermostable direct hemolysin (TDH) encoded by *tdh* and TDH-related hemolysin (TRH) encoded by *trh* are two major virulence factors leading to *V. parahaemolyticus*-mediated disease (Nishibuchi and Kaper, 1995). However, the *trh* gene has two variants (*trh1*, *trh2*), which only share 84% sequence identity (Kishishita et al., 1992; Raghunath et al., 2010; Niu et al., 2018). Classification errors are emerged due to the lack of sequence identity of the *trh* gene and the relatedness of the hemolysin toxins (Suthienkul et al., 1995; Niu et al., 2018). The *trh* gene cluster is immediately downstream of *ureR*, so these two genes are genetically-linked. Using a more highly conserved *ureR* gene as a target is a useful tool for proxy detection of *trh* and can overcome difficulties associated with *trh* variation (Nilssona and Turner, 2016). The *V. parahaemolyticus* pandemic group strains including O3:K6 and its derivatives (the O4:K68, O1:K25, and O1:KUT serotypes) have emerged since 1996 (Matsumoto et al., 2000; Han et al., 2015). The *orf8* gene, a peculiar open reading frame of the filamentous phage f237, is their common genetic marker (Nasu et al., 2000; Myers et al., 2003). A putative adherence protein encoded by *orf8* may increase the virulence of O3:K6 (Ceccarelli et al., 2013; Letchumanan et al., 2014). Therefore, the *tlh*, *tdh*, *ureR*, and *orf8* genes are generally used as targets to determine whether a strain is pathogenic *V. parahaemolyticus* (Su and Liu, 2007; Letchumanan et al., 2014).

**FIGURE 1:**
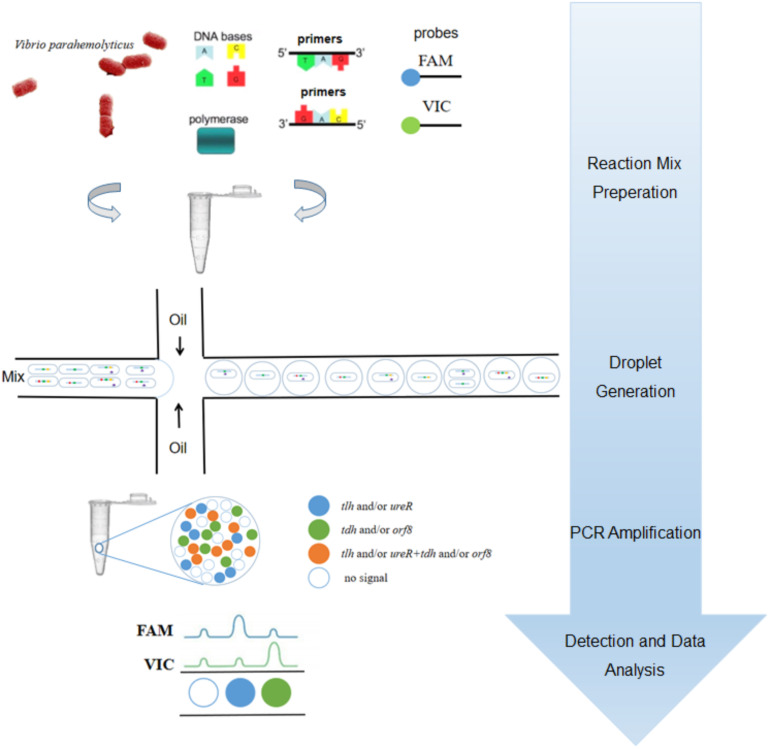
The procedural framework of the 4-plex ddPCR for specific detection of intact *V. parahaemolyticus* cells. The bacterial solution containing intact cells were added to the PCR reaction. The cells were dispersed into droplets prior to PCR-amplification. The targets of *tlh* (green), *tdh* (red), *ureR* (yellow), and *orf8* (purple) were determined based on fluorescent probes.

To our knowledge, most researchers have developed qPCR for detecting *V. parahaemolyticus* (Su and Liu, 2007; Letchumanan et al., 2014; Zhang et al., 2015a,b). However, there were few studies on the detection of *V. parahaemolyticus* based on ddPCR. In previous work, we have developed a 3-plex ddPCR method for detecting and genotyping *V. parahaemolyticus* (Lei et al., 2020). However, the *orf8* gene is not included in the study. In this paper, we established a 4-plex ddPCR method for the first time which could detect *V. parahaemolyticus* with single intact cells, as illustrated in Figure 1. In brief, the suspension of bacterial cells in ddPCR mixture was dispersed into droplets with a microfluidic chip, followed by the release of gDNA by hot lysis, ddPCR reaction and analysis. As four probes of these genes were labeled by only two fluorescent dyes, the probe concentrations were proportionally distributed to form a matrix with 16 (2^4^) clusters, which explicitly displayed and quantified droplets containing one/two/three/four certain genes, corresponding to gene types of single intact bacterial cells. This method was beneficial for identifying *V. parahaemolyticus* encoded by different virulence genes accurately, rapidly, selectively, sensitively and reproducibly, which could offer precise supports to clinical diagnosis and treatments in bromatoxism, as well as in the monitoring of seafood contamination.

## Materials and Methods

### Bacterial Strains and Culture Conditions

The strains used in this study are shown in Table 1. All *Vibrio* strains were cultured in tryptic soy broth (TSB; Beijing Land Bridge Technology Company Ltd., Beijing, China) with 3% (w/w) sodium chloride (NaCl) on a rotary shaker (YiHeng, ShangHai, China) at 150 rpm and 37°C for 16 h. All other strains were cultured in Luria-Bertani (LB) broth and kept in a shake incubator (150 rpm) at 37°C for 16 h. The bacterial cells were 10-fold serial diluted for ddPCR detection. The plate count method was used for comparing with the ddPCR method, where bacteria were grown on TCBS (hopebio, QingDao, China) or LB agar plates at 37°C for 24 h.

**TABLE 1:** Specificity of the ddPCR for *tlh*, *tdh*, *ureR*, and *orf8* target genes for different bacterial strains.

Bacterial species	Strains	ddPCR results			
		*tdh*	*tlh*	*ureR*	*orf8*
*V*. *parahaemolyticus*	ATCC 17802	+	+	+	–
*V. parahaemolyticus*	J5421	+	+	–	+
*V. parahaemolyticus*	SCAUFS VP1	+	+	–	–
*V. parahaemolyticus*	SCAUFS VP2	–	+	–	–
*V. parahaemolyticus*	SCAUFS VP3	–	+	+	–
*V. parahaemolyticus*	SCAUFS VP5	–	+	–	–
*V. parahaemolyticus*	SCAUFS VP7	+	+	–	–
*V. parahaemolyticus*	SCAUFS VP10	+	+	–	–
*V. parahaemolyticus*	SCAUFS VP11	–	+	–	–
*V. parahaemolyticus*	SCAUFS VP12	+	+	–	–
*V. parahaemolyticus*	SCAUFS VP13	–	+	+	–
*V. parahaemolyticus*	SCAUFS VP14	–	+	–	–
*V. parahaemolyticus*	SCAUFS VP15	+	+	–	–
*V. parahaemolyticus*	SCAUFS VP16	–	+	–	–
*V. parahaemolyticus*	SCAUFS VP17	+	+	–	–
*V. parahaemolyticus*	SCAUFS VP18	–	+	–	–
*Vibrio alginnolyficus*	SCAUFS 4	+	–	–	–
*Vibrio vulnificus*	SCAUFS 6	–	–	–	–
*Vibrio vulnificus*	SCAUFS 9	–	–	–	–
*V. shewanella*	SCAUFS 5001	–	–	–	–
*V. azureus*	SCAUFS 5019	–	–	–	–
*Bacillus subtilis*	CGMCC 1.4255	–	–	–	–
*B. subtilis*	CGMCC 1.3358	–	–	–	–
*B. thuringiensis*	CGMCC 1.1013	–	–	–	–
*Escherichia coli*	CGMCC 1.2835	–	–	–	–
*E. coli*	CMCC 44102	–	–	–	–
*E. coli* O157:H7	SCAUVM 3001	–	–	–	–
*Shigella dysenteriae*	CGMCC 1.1869	–	–	–	–
*Staphylococcus aureus*	CMCC 26003	–	–	–	–
*S. aureus*	CGMCC 1.2465	–	–	–	–
*Salmonella enterica*	CGMCC 1.1194	–	–	–	–
*S. enterica*	CGMCC 1.10603	–	–	–	–
*S. typhimurium*	CMCC 50115	–	–	–	–
*Listeria monocytogenes*	ATCC 19115	–	–	–	–
*L. monocytogenes*	CGMCC 1.9144	–	–	–	–
*Lactococcus lactis*	CGMCC 1.2470	–	–	–	–
*Lactobacillus delbrueckii*	GIM 1.155	–	–	–	–
*Lactobacillus plantarum*	ATCC 8014	–	–	–	–

*ATCC, American Type Culture Collection; CGMCC, China General Microbiological Culture Collection Center; SCAUFS, Food Safety Lab of South China Agricultural University; CMCC, China Medical Culture Collection; SCAUVM, Veterinary Medicine Lab of South China Agricultural University; GIM, Guangdong Institute of Microbiology.*

### Primers and Probes

According to *V. parahaemolyticus* strain ATCC17802 (CP014046.2, CP014047.2) and *V. parahaemolyticus* plasmid pO3K6 DNA (NC_002473.1; *tdh*, *tlh*, and *trh/ureR* are located in chromosome 2, *orf8* is usually located in plasmid), multiplex primers and probe sets for *tlh*, *tdh*, *ureR*, and *orf8* genes were designed by the Primer Premier 5.0 (Premier Biosoft, Canada). Specificity analysis of amplicons was carried out using nucleotide BLAST (National Center for Biotechnology Information, Bethesda, MD, United States). The sequences of the primers and probes in this study were listed in Table 2.

**TABLE 2:** Primers and probe sequences used in the 4-plex ddPCR.

Taget	Primer	5′-sequence-3′	Product size (bp)
*tlh*	*tlh*-F	GAACGCAGACATTACG	105
	*tlh*-R	ACCACTTTGTTGATTTGA	
	*tlh*-P	FAM-CATTGCTGCGTCGTTGCTCC-BHQ1	
*tdh*	*tdh*-F	GGTCAGGAAGTTCGT	121
	*tdh*-R	ACGGCATAGGTGAGTA	
	*tdh*-P	VIC-CCGCCACGACAGTTACGA-BHQ2	
*ureR*	*ureR*-F	GCACTCTAACACCCAA	111
	*ureR*-R	AGCTGATACATCGGTT	
	*ureR*-P	FAM-CTAGGCGAGCAAAAGCACTCT-BHQ1	
*orf8*	*orf8*-F	GCACCCTAAACAAAA	162
	*orf8*-R	AGAGGTACAAGATCA	
	*orf8*-P	VIC-CCCCACGACAGCC-BHQ2	

### Optimization of the Sample Hot Lysis Time and the Cycle Number

In order to increase the detection accuracy and effectiveness, both the sample hot lysis time and the cycle number were optimized. Four sample hot lysis time lengths (15, 20, 25, and 30 min) and four different cycle numbers (45, 50, 55, and 60 cycles) were tested, respectively.

### Quantitative PCR

The qPCR reaction was conducted in a total reaction volume of 20 μL containing 0.5 μM of each primer F/R and 0.125 μM of the probe, 10 μL of 2 × SGExcel GoldStar TaqMan Mixture (Sangon Biotech, Shanghai, China), 2 μL of cell template, and nuclease-free distilled water up to the final volume. The reaction process started by hot lysis treatment at 95°C for 30 min, followed by 60 cycles of 30 s at 95°C for denaturation, 1 min at 54°C for annealing and extension.

### 4-plex Droplet Digital PCR

Droplet digital PCR (ddPCR) was carried out on the MicroDrop^TM^ (MD) ddPCR system (Forever Gene, Shunde, Guangdong, China), which was consist of Microchip, Creator, and Detector for microdrop operations. The ddPCR mixture was performed in a 20 μL reaction volume composed of 2 μL of cell suspension, 10 μL of 2 × Supermix for Probes (Forever Gene), 900 nM of each primer, 0.125 μM of *tlh*, 0.625 μM of *tdh*, 0.25 μM of *ureR*, and 1.25 μM of *orf8* probes, nuclease-free distilled water up to the final volume. The cell suspensions were serially diluted to ensure the majority of droplets were ideally either contained single cell or empty. Approximate 80 thousand water-in-oil droplets were produced using MD Microchip and MD Creator, then transferred to a conventional 96-well PCR plate followed by heat-sealing. In each droplet, an independent reaction was conducted to end point using the ABI Veriti FAST PCR (United States). The protocol was as follow: 25 min at 95°C for the cell hot lysis and Taq hot start, followed by 55 cycles of 30 s at 95°C (denaturation), 60 s at 54°C (annealing and extension), and a final hold of 10 min at 98°C (droplet stabilization), then cooling to 25°C. Following amplification, the samples were transferred to a MD Detector (Forever Gene), and the fluorescence amplitudes of four genes in every cell were read. The MD software then fit the fraction of positive droplets to the Poisson distribution algorithm to determine the number of targets and identify different strains of *V. parahaemolyticus.*

### Comparing ddPCR With Plate Counting and qPCR

To evaluate the sensitivity of the 4-plex ddPCR assays, the same samples were simultaneously detected by plate counting, qPCR, and ddPCR. The 10-fold serially diluted *V. parahaemolyticus* solutions (3.9 × 10^1^–3.9 × 10^7^ CFU/mL) were grown on TCBS agar plates at 37°C for 24 h, as well as 2 μL solution of each sample was detected directly by qPCR and ddPCR. All assays were triplicated and repeated for three times. The standard curves were constructed using ten-fold serial dilutions of the mixed bacterial solution containing *V. parahaemolyticus* ATCC17802 and *V. parahaemolyticus* J5421 (2:1) covering the range from 3.9 × 10^7^ to 3.9 × 10^1^ CFU/mL.

### Assessment of the 4-plex ddPCR Method With Complex Cell Templates

In this assay, eight types of cell templates (genes) were prepared for the 4-plex ddPCR evaluations for mimic complex detections: template 1 (*tlh*), template 2 (*tlh* and *ureR*), template 3 (*tlh* and *tdh*), template 4 (*tlh* and *orf8*), template 5 (*tlh*, *ureR*, and *tdh*), template 6 (*tlh*, *tdh*, and *orf8*), template 7 (*tlh*, *ureR*, and *orf8*), and template 8 (*tlh*, *tdh*, *ureR*, and *orf8*).

### Detection of *V. parahaemolyticus* in the Background of High Concentrations of Non-target Bacteria

To study the impact of high levels of non-target bacteria (*Escherichia coli* and *Listeria monocytogenes*), mixtures of *V. parahaemolyticus* (3.9 × 10^3^ CFU/mL) with different concentrations (10^1^–10^7^ CFU/mL) of non-target bacteria were prepared. In this assay, the *V. parahaemolyticus* at 3.9 × 10^3^ CFU/mL was chosen as control. Meanwhile, the suspensions containing 10^6^ CFU/mL of non-target bacteria and *V. parahaemolyticus* cells at different concentrations (3.9 × 10^1^–3.9 × 10^7^ CFU/mL) were prepared for detection of *V. parahaemolyticus* at serial concentrations in the background of high levels of certain non-target bacteria.

### Verifying Assay With Artificial Contaminated White Calms

White clams were obtained from supermarket and washed by ddH_2_O. Twenty-five grams of clam tissues were inoculated with 1 mL bacterial solution at a concentration of 10^8^ CFU/g and mixed with 225 mL 0.1% aseptic peptone water (PW). Then the mixture was homogenized for 5 min and centrifuged for 1 min (1000 rpm, 4°C). The supernatant was filtered to remove large particles and centrifuged at 12,000 rpm for 10 min to obtain the bacterial cells. Finally, the bacterial cells were suspended in 250 μL ddH_2_O and were serially diluted as templates for the 4-plex ddPCR.

## Results

### Specificity of Primers and Probes

Ten strains of *V. parahaemolyticus* and 22 strains of non-*V. parahaemolyticus* were tested to determine the specificity of the 4-plex ddPCR assays. As shown in Table 1, only the target bacterial strains are positively observed, while no positive signal occurred for non-target strains, confirming 100% inclusivity and exclusivity for *V. parahaemolyticus* by these assays. Here we know that strain ATCC17802 is *tlh^+^tdh^+^ureR^+^*, and strain J5421 is *tlh^+^tdh^+^orf8^+^*. These two strains were adopted as sample strains in subsequent works.

### Optimization of the Heat Lysis Time of the Sample

**FIGURE 2:**
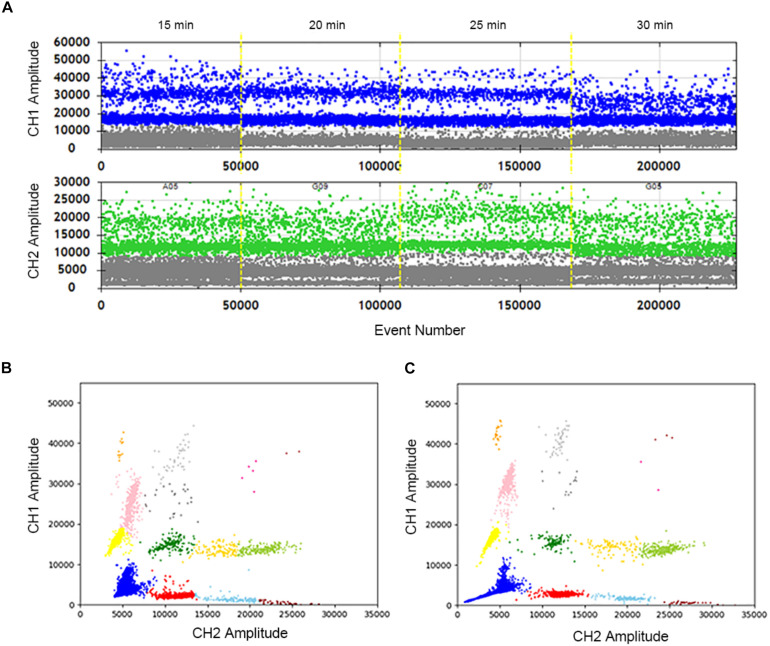
Optimization of the sample hot lysis time in two channels and 2D plot. **(A)** The positive droplets were represented as blue and the negative droplets were represented as gray in FAM. The positive droplets were represented as green and the negative droplets were represented as gray in VIC. **(B)** The 2D plot was obtained in 30 min of the hot lysis time. **(C)** The 2D plot was obtained in 25 min of the sample hot lysis time.

The samples were treated at 95°C for different time lengths: 15, 20, 25, and 30 min. When the heat lysis time is 20 min or 25 min, positive and negative droplets are well separated. The fluorescence amplitude is highest at 25 min (Figure 2A). Compared with 30 min (Figure 2B), every cluster is concentrated and can be well distinguished at 25 min (Figure 2C). In summary, the sample treated at 95°C for 25 min displays superior 2D plots.

### Optimization of Cycle Number

**FIGURE 3:**
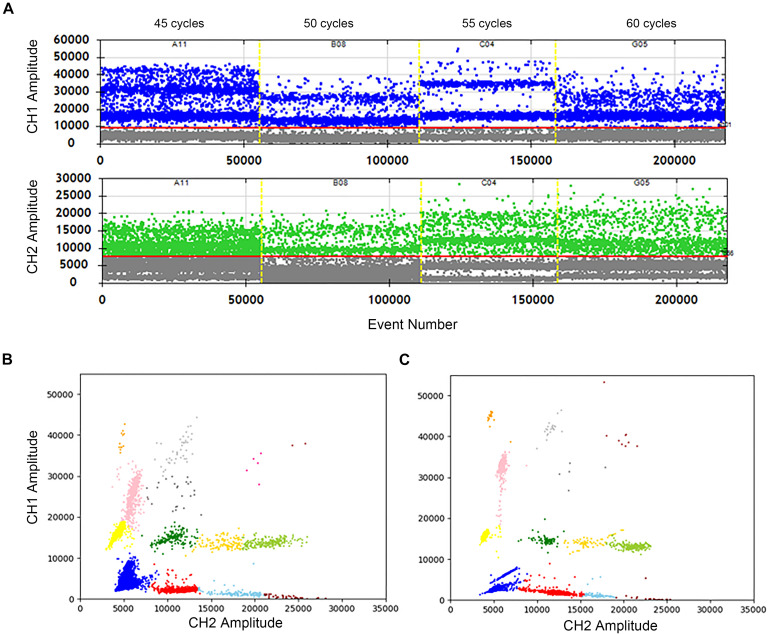
Optimization of the cycle number in two channels and 2D plot. **(A)** The positive droplets were represented as blue and the negative droplets were represented as gray in FAM. The positive droplets were represented as green and the negative droplets were represented as gray in VIC. **(B)** The 2D plot was obtained in 60 cycles of the amplification. **(C)** The 2D plot was obtained in 55 cycles of the amplification.

The effects of cycle numbers at 45, 50, 55, and 60 were investigated. In Figure 3A, the vertical axis shows the fluorescence amplitude values. In the FAM channel (CH1), significant differences among 4 wells are observed. The total droplet number of the four wells are similar, indicating that cycle numbers don’t impact the stabilization of droplets. However, when the cycle number is 55, the clusters are divided clearly with high fluorescence amplitudes, and droplets in the “rain” between clusters are least. The similar conclusions can easily be drawn, which shows the highest fluorescence amplitude and separated clusters in the HEX/VIC channel (CH2). Meanwhile, a comparison of the 2D plot of 60 cycles (Figure 3B) with that of 55 cycles (Figure 3C) illustrate that the clusters are separated more completely, droplets in the “rain” decrease and the fluorescence amplitude increases in the condition of 55 cycles. Therefore, 55 cycles would be better for this assay. Hence, not only detection time is reduced, but also the accuracy is improved after optimizations.

### Performance of Plate Counting, qPCR and ddPCR

**FIGURE 4:**
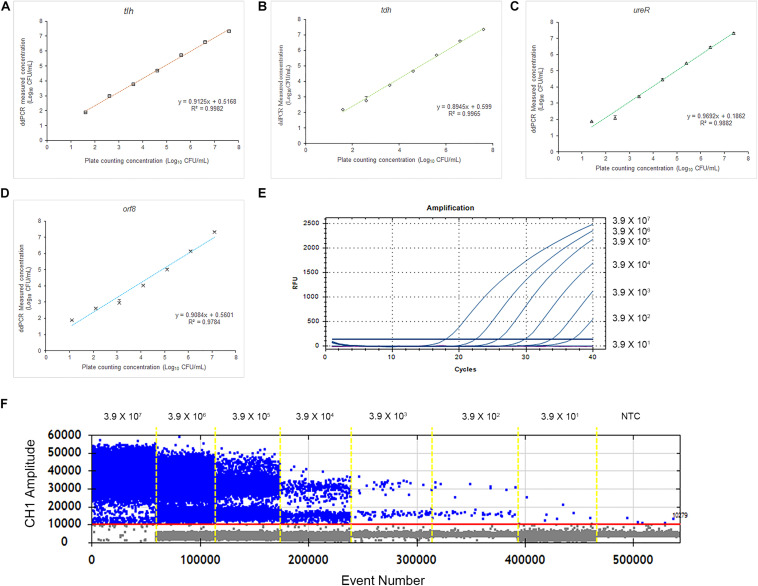
Quantification of *V. parahaemolyticus* by plate counting, qPCR, and ddPCR. **(A)** Standard curves of *tlh* in 4-plex ddPCR. Comparative analysis of diluted series of indicated bacterial solution. The horizontal axis was plate counting detection results for *V. parahaemolyticus.* **(B)** Standard curves of *tdh* in 4-plex ddPCR. **(C)** Standard curves of *ureR* in 4-plex ddPCR. **(D)** Standard curves of *orf8* in 4-plex ddPCR. **(E)** Fluorescence curves were obtained based on qPCR by using the same templates. **(F)** The picture of FAM channel of the 4-plex ddPCR by using the same templates. Plotted values represented the mean value and standard deviations obtained from three triplicate tests. NTC = non-template control.

To evaluate the accuracy of the 4-plex ddPCR assays, we compared this method with the standard plate counting method. The 10-fold diluted *V. parahaemolyticus* solutions (3.9 × 10^1^–3.9 × 10^7^ CFU/mL) were detected for plate counting, qPCR and ddPCR. As shown in Figures 4A–D, the results of plate counting on TCBS agar are agreed with ddPCR results ranging from 3.9 × 10^7^–3.9 × 10^1^ CFU/mL, illustrating a good linear relationship of the detection with a correlation coefficient (R^2^) of 0.9982 (*tlh*), 0.9965 (*tdh*), 0.9882 (*ureR*), and 0.9784 (*orf8*), respectively. We noted that absolute quantifications by ddPCR corresponded to 87.0–137.1% of the theoretical cell numbers by plate counting, indicating that absolute detection by ddPCR is credible. Additionally, the measured concentration by ddPCR is always higher than that of the traditional plate counting method. It exhibited that our 4-plex ddPCR provided an accurate and efficient tool for the detection of *V. parahaemolyticus*. Figures 4E,F present the typical results of detecting different concentrations of *V. parahaemolyticus* based on the ddPCR and qPCR. It indicates that when the concentration of *V. parahaemolyticus* decreases to 3.9 × 10^1^ CFU/mL, the fluorescence curve of qPCR disappears; meanwhile, the corresponded data of ddPCR can be presented, and the limit of detection (LOD) of ddPCR is defined as 39 CFU/mL. Compared with qPCR, the ddPCR method showed greater potential for sensitive detection of *V. parahaemolyticus*, especially at lower concentrations.

### 4-plex ddPCR Detecting Different Strains of *V. parahaemolyticus*

**FIGURE 5:**
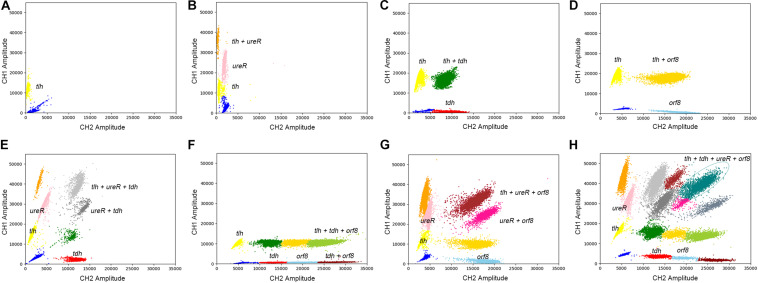
The 2D plot of different targets, the target contains **(A)** *tlh*; **(B)** *tlh*, *ureR*; **(C)** *tlh*, *tdh*; **(D)** *tlh*, *orf8*; **(E)** *tlh*, *ureR*, and *tdh*; **(F)** *tlh*, *tdh*, and *orf8*; **(G)** *tlh*, *ureR*, and *orf8*; **(H)** *tlh*, *tdh*, *ureR*, and *orf8*.

The results of eight detection systems using different templates were shown in Figure 5. It has to be underlined that a better quality in terms of clusters separation had been obtained after optimizations. Moreover, when the templates are positive for *tlh*, *tdh*, *ureR*, and/or *orf8*, the specific clusters are presented in the 2D-plot. According to the cluster position, we could directly distinguish the bacteria with different genes. Therefore, the 4-plex ddPCR is a feasible and accurate method for the classification of different strains of *V. parahaemolyticus*.

### Distinguishing *V. parahaemolyticus* From High Levels of *Escherichia coli* and *Listeria monocytogenes*

**FIGURE 6:**
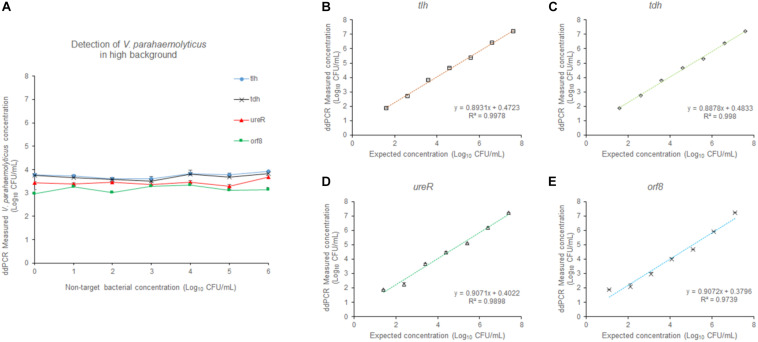
The effect of the high concentration of non-target bacteria on the detection. **(A)** At different concentrations (10^1^–10^6^ CFU/mL) of non-target bacteria, *V. parahaemolyticus* at the concentration of 10^3^ CFU/mL was detected. At the concentration (10^6^ CFU/mL) of non-target bacteria, *V. parahaemolyticus* samples containing *tlh* **(B)**, *tdh* **(C)**, *ureR* **(D)**, *and orf8* **(E)** at different concentrations (10^1^–10^7^ CFU/mL) were detected. Plotted values represented the means of 3 independent replicates.

In order to evaluate the selectivity of the 4-plex ddPCR assay for *V. parahaemolyticus* detection, other pathogenic bacteria including *E. coli* and *L. monocytogenes* at a concentration from 10^1^–10^6^ CFU/mL, were examined under the same detecting conditions. The *V. parahaemolyticus* cultures were diluted to approximately 3.9 × 10^3^ CFU/mL. Figure 6A reveals that the measured concentrations by our method were close to that of the control, while background bacteria are at different concentrations (10^1^–10^6^ CFU/mL). The results indicate that the 4-plex ddPCR method displays good selectivity for *V. parahaemolyticus*, and the detections of *V. parahaemolyticus* are not disturbed by other bacteria no matter how high the non-target bacteria concentrations (10^1^–10^6^ CFU/mL) are. In Figures 6B–E, when the concentration of background bacteria is 10^6^ CFU/mL, the detections of *V. parahaemolyticus* (3.9 × 10^1^–3.9 × 10^7^ CFU/mL) have a good linear and are in consistent with the results without background bacteria. The R^2^ are 0.9978 (*tlh*), 0.998 (*tdh*), 0.9898 (*ureR*), and 0.9739 (*orf8*). The results indicate that either in the absence or presence of high concentrations of non-target bacteria, the detections of *V. parahaemolyticus* at different concentrations (10^1^–10^7^ CFU/mL) are not impacted.

### Performance of the 4-plex ddPCR Assay in Detecting Artificial Contaminated Seafood Samples

**FIGURE 7:**
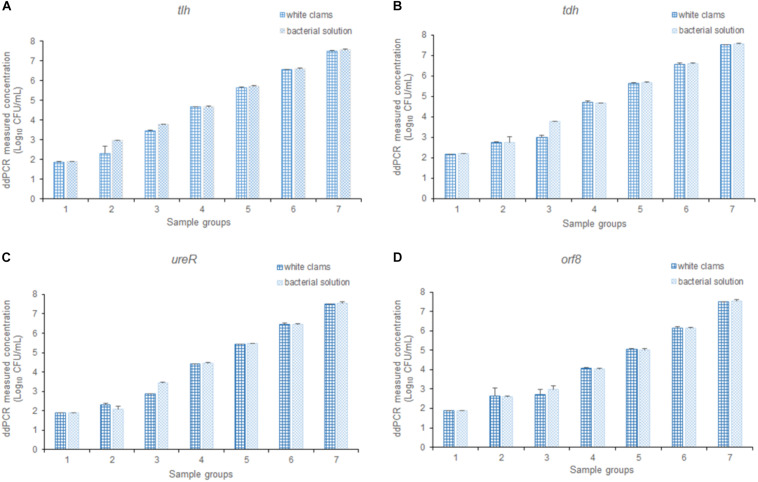
The detection results of *V. parahaemolyticus* in solution and spiked white clams, analyzing **(A)** *tlh*, **(B)** *tdh*, **(C)** *ureR*, and **(D)** *orf8*, respectively. Plotted values represented the mean value and standard deviations obtained from three triplicate tests.

To assess the efficiency of the multiplex ddPCR, both quantitative bacterial solution and artificial contaminated white clam tissue containing the equal concentration of bacterial cells were used as the templates for the detection of *V. parahaemolyticus*. As shown in Figure 7, there is no significant difference between the results of the two templates (*p* > 0.05). It confirmed that the established method was applicable for the determination of *V. parahaemolyticus* in a wide range of concentrations in complex food samples.

## Discussion

*Vibrio parahaemolyticus* is an enteric pathogen found in a variety of seafood, especially clams, shrimps, and oysters (Liu et al., 2018; Cao et al., 2019). Although the presence of *V. parahaemolyticus* is extensive in marine and estuarine environments, not all strains of *V. parahaemolyticus* result in illness (Colwell et al., 1977). Only a small subset of *V. parahaemolyticus* strains are pathogenic (Velazquez-Roman et al., 2014). In molecular epidemiological studies, the strains with *tdh*^+^, and/or *trh*^+^ were generally distinguished as virulent strains, which were up to 90% of the isolated clinical strains (Zhang and Austin, 2005; Chao et al., 2010). Either TDH, TRH, or both can be produced by pathogenic *V. parahaemolyticus* (Honda and Iida, 1993; Nishibuchi and Kaper, 1995). Hence, the target genes *tdh* and *trh*, encoding TDH hemolysin and TRH hemolysin, respectively, have been used for the classification of pathogenic isolates of *V. parahaemolyticus* (Shirai et al., 1990; Yoh et al., 1995). The transcriptional regulator for the urease gene cluster was encoded by the *ureR* gene, immediately upstream of *trh*. Replacing *trh* with *ureR* as a target gene could circumvent *trh* sequence variation (Nilssona and Turner, 2016). In February 1996, an atypical increase in diarrheal infections caused by *V*. *parahaemolyticus* occurred in Kolkata and India. Then, this clone rapidly spread throughout the majority of Southeast Asian, Atlantic, and Gulf coasts of the United States (Okuda et al., 1997; Chowdhury et al., 2000; Matsumoto et al., 2000), Europe (Martinez-Urtaza et al., 2005), and Africa (Ansaruzzaman et al., 2005). This epidemiology was related to *V. parahaemolyticus* O3:K6 serotype, which was pandemic strain generally contain the *orf8* gene. All in all, it is of great importance to apply *tlh*, *tdh*, *ureR*, and *orf8* to facilitate accurate quantification of *V. parahaemolyticus* in samples. Based on this method, we can take more genes such as *trh*, *toxR*, and *toxS* into account in the future.

What kind of genes the bacteria carried determined the bacterial characteristics. The 4-plex ddPCR method was very effective for detecting *tlh*, *tdh*, *ureR*, and *orf8* genes simultaneously. It was beneficial to find linkage among the 4 targets for classifying *V. parahaemolyticus*. When gDNA extracts were used as the template, the fragments would be segregated into different droplets. However, when the intact cell was used as a template, a single cell was contained in a droplet and the linkage among four genes in one cell was presented. In the previous report, the multiplexed single intact cell droplet digital PCR (MuSIC ddPCR) method was developed for the detection of Enterohemorrhagic *E. coli* (EHEC; McMahon et al., 2017). However, it was only 2-plex and displayed low amplification efficiency. In this paper, the 4-plex ddCR method based on the single intact cell for the detection of *V. parahaemolyticus* was first established. The results indicated that our method not only could identify pathogenic *V. parahaemolyticus* conveniently, precisely and reliably, but also was more sensitive than qPCR.

For determining the influence of non-target bacteria, two experiments were conducted. On one hand, *V. parahaemolyticus* (3.9 × 10^3^ CFU/mL) mixed with different concentrations of non-target bacteria (10^1^–10^6^ CFU/mL) were detected. On the other hand, different concentrations (3.9 × 10^1^–3.9 × 10^7^ CFU/mL) of *V. parahaemolyticus* in the high background of non-target bacteria (10^6^ CFU/mL) were tested. The *V. parahaemolyticus* detection results of the samples with non-target bacteria were consistent with that of the sample without non-target. The presence of non-target bacteria showed less impact on detection. The result presented strong evidence that our method displayed high sensitivity and specificity. Different from other ddPCR detection which had narrow range (Wang et al., 2018; Zhong et al., 2018; Jahne et al., 2019; Nshimyimana et al., 2019), our method presented a wider detection range from 3.9 × 10^7^ to 3.9 × 10^1^ CFU/mL. Owing to approximate 80 thousand droplets produced, a narrowed detection range of ddPCR was circumvented. In addition, our detection method required a small amount of sample (2 μL), which provided new ideas for the detection of rare samples.

## Conclusion

In conclusion, the 4-plex ddPCR method was developed for the rapid, sensitive, convenient, and reliable detection of *V. parahaemolyticus*. Four characteristic target genes of *V. parahaemolyticus* including *tlh*, *tdh*, *ureR*, and *orf8* were successfully determined. It could not only absolutely quantify *V. parahaemolyticus* cells but also effectively help classify different strains of *V. parahaemolyticus*. This 4-plex ddPCR method would greatly facilitate the detection of different serotypes of *V. parahaemolyticus* and provide a new method for detecting other bacteria.

## Data Availability Statement

The raw data supporting the conclusions of this article will be made available by the authors, without undue reservation, to any qualified researcher.

## Author Contributions

SL performed the experiments and prepared the manuscript. XG designed the experiments and analyzed the data. WX contributed to manuscript revision. ZR contributed analysis tools. ZW provided reagents. SC performed parts of the experiments. QZ designed the experiments and contributed to manuscript writing. All authors contributed to the article and approved the submitted version.

## Conflict of Interest

The authors declare that the research was conducted in the absence of any commercial or financial relationships that could be construed as a potential conflict of interest.
